# Epidemiology of companion animal AMR in the United States of America: filling a gap in the one health approach

**DOI:** 10.3389/fpubh.2023.1161950

**Published:** 2023-06-16

**Authors:** Kurtis E. Sobkowich, J. Scott Weese, Zvonimir Poljak, Andy Plum, Donald Szlosek, Theresa M. Bernardo

**Affiliations:** ^1^Department of Population Medicine, Ontario Veterinary College, University of Guelph, Guelph, ON, Canada; ^2^Centre for Public Health and Zoonoses, Ontario Veterinary College, University of Guelph, Guelph, ON, Canada; ^3^Department of Pathobiology, Ontario Veterinary College, University of Guelph, Guelph, ON, Canada; ^4^IDEXX Laboratories, Inc., Westbrook, ME, United States

**Keywords:** antimicrobial resistance, *E. coli*, *S. pseudintermedius*, epidemiology, companion animal

## Abstract

**Introduction:**

Antimicrobial resistance (AMR) is a global health concern that affects all aspects of the One Health Triad, including human, animal, and environmental health. Companion animals, such as cats and dogs, may contribute to the spread of AMR through their close contact with humans and the frequent prescription of antimicrobials. However, research on AMR in companion animals is limited, and there are few surveillance measures in place to monitor the spread of resistant pathogens in the United States.

**Methods:**

This study aims to explore the practicality of using data from commercial laboratory antimicrobial susceptibility testing (AST) services for epidemiological analyses of AMR in companion animals in the United States.

**Results:**

The study analyzed 25,147,300 individual AST results from cats and dogs submitted to a large commercial diagnostic laboratory in the United States between 2019 and 2021, and found that resistance to certain antimicrobials was common in both *E. coli* and *S. pseudintermedius* strains.

**Conclusion:**

There has been a paucity of information regarding AMR in companion animals in comparison to human, environmental and other animal species. Commercial AST datasets may prove beneficial in providing more representation to companion animals within the One Health framework for AMR.

## 1. Introduction

Antimicrobial resistance (AMR) is a primary global health concern, affecting all fractions of the One Health Triad: human, animal, and environmental health (1). Resistance can also be transferable between human and animal species, making AMR a zoonotic health concern as well (2, 3). Articles stressing the importance of a One Health approach to AMR often neglect the role of companion animals, failing to account for a missing piece of the puzzle.

Growing awareness of the potential contribution of companion animals to the spread of resistant pathogens has led to a greater research focus on household cats and dogs due to the intimate bond shared with their owners. Frequent close contacts present the opportunity for transmission of antimicrobial resistant bacteria (4), but are rooted in a deeply held bond owners share with their animals (5). Additionally, companion animals share more antimicrobials and are treated for infections in a manner more similar to humans than livestock animals, presenting greater opportunity for resistant pathogen spillover (6). However, data on companion animal AMR are typically less abundant than that of production animals. The American Food and Drug Administration recently recognized the absence of research into companion animal AMR and the immediate need to fill this gap in the One Health approach (7).

In the United States, it is estimated that one in five households acquired a new cat or dog during the COVID-19 pandemic (8), adding to the previous 135 million pets owned in 2018 (9). As this new generation of cats and dogs age, there will be an inevitable demand for antimicrobial treatments. Coupled with current resistance concerns, this demand may lead to the propagation of new and existing strains of resistant microbes within the next 15 years, for which surveillance measures must be put into place. For such surveillance, sufficient data must be collected continuously over a broad geographic area. Routine susceptibility testing by diagnostics laboratories may provide an avenue to achieve this level of data collection, but the usefulness of such data sources for epidemiological studies of companion animal AMR has yet to be adequately assessed due to limited access. Previous works have leveraged electronic health records from companion animals to develop a passive surveillance system for tick monitoring (10), and such methodologies may be applicable for AMR surveillance, but first data sources must be explored.

An estimate of the global burden of antimicrobial resistance in 2019 found over 5 million human deaths attributable to AMR, 75% of them being accounted for by only six pathogens, with *Escherichia coli* and *Staphylococcus aureus* being at the top of the list (11). Resistant *S. aureus* represents a major health concern for humans but is not as commonly isolated in cats and dogs compared to *S. pseudintermedius*, which can commonly be misidentified as *S. aureus* in human animal bites (12). *S. pseudintermedius* is an underreported issue in human medicine, but awareness is increasing due to its similarities to other human pathogens (13, 14).

This study aims to highlight the underrepresentation of companion animals in the current One Health framework of antimicrobial resistance. Companion animals are often neglected from public health reports on AMR due to the inaccessibility of data in comparison to humans, and livestock (15). However, routine commercial antimicrobial susceptibility testing may offer a wide-reaching and continuing source of data for epidemiology and surveillance. This study demonstrates the application of these types of data. Given the complexity of antimicrobial resistance, it is not feasible to represent all drug-pathogen resistance combinations in a single study. Therefore, a selection of clinically relevant *E. coli* and *S. pseudintermedius* resistance concerns are investigated, to provide a high-level overview of the status of known and emerging resistance concerns, using a previously unexploited source of data.

## 2. Materials and methods

Samples from cats and dogs submitted to a nation-wide commercial diagnostic laboratory within the United States for bacteriological testing and subsequent antimicrobial susceptibility testing between 2019 and 2021 were eligible for inclusion. Each observation was recorded with the following information: a unique deidentified patient number, the date of sample collection (down to monthly accuracy), the location of the submitting veterinary practice (three-digit ZIP code accuracy), the source from which the sample was taken on the animal, the identified pathogen isolated in the sample, and the antimicrobial susceptibility status for all drugs tested against the isolated pathogen. Susceptibility status was reported as either “susceptible,” “resistant,” or “intermediate” based on clinical breakpoint values set forth by the Clinical and Laboratory Standards Institute (CLSI) (16). Duplicated samples were defined as possessing the same identification number, same isolated pathogen, occurring within the same three-month quarter and the same susceptible/intermediate/resistant status. Duplicates were removed. All susceptibility testing was conducted on the VITEK (bioMérieux) automated platform (17). Where VITEK testing was not possible, the Kirby-Bauer method was used (18). Canine and feline data were assessed in aggregate.

Four drug-pathogen susceptibility scenarios were investigated, including two representing common first line prescription approaches and two emerging resistance concerns of higher priority drugs. The specific drug-pathogen combinations included: amoxicillin resistant *Escherichia coli* isolated from urine samples, cephalexin (first-generation cephalosporin) resistant *S. pseudintermedius* isolated from skin samples, third-generation cephalosporin resistant *E. coli* (any source), and methicillin resistant *S. pseudintermedius* (any source). Third-generation cephalosporin resistance was indicated primarily by cefotaxime resistance. Where cefotaxime was not tested but another third-generation cephalosporin was (i.e., ceftazidime or cefpodoxime), that antimicrobial would be included in its place, with a limit of one drug per sample. Oxacillin was used to test for methicillin resistance.

Choropleth maps representing the percentage of samples found to be non-susceptible were produced for each of the four drug-pathogen combination scenarios at the state level. Non-susceptibility was defined as a sample either being reported as resistant or intermediately resistant. State borders were acquired from the ‘*usmaps*’ package in the statistical software *R* (19, 20). Where applicable, Wald 95% confidence intervals were used given the large sample size. The data were then further stratified into individual calendar years, and relative risk values for each state were calculated using the following formula: proportion of resistant samples within the state divided by the proportion of resistant samples in all the United States for a given year. This metric provided a standardized measure to indicate the deviation of a given state from the nationally expected amount of resistance, with values less than 1 indicating less than expected resistance, values greater than 1 indicating greater than expected resistance and a value of 1 indicating equal to the expected resistance. These relative risk values were mapped and arranged in chronological order for each drug-pathogen scenario to provide indication of any emerging temporal patterns. In both maps, information on any state with less than 30 sample observations was censored as per CLSI’s report ‘Analysis and Presentation of Cumulative Antimicrobial Susceptibility Test Data, 5th Edition’ (21).

To compare the level of resistance between drugs of the same class against a common pathogen, the proportion of susceptible, resistant and intermediate interpretations were tabulated for all the tested third generation cephalosporins and fluoroquinolones against *E. coli* or *Staphylococcus* spp. (*S. aureus, S. intermedius, S. pseudintermedius, S. schleiferi*). All samples of *E. coli* or *Staphylococcus spp.* submitted for susceptibility testing were included, regardless of repeated testing. These tabulated proportions of test interpretations provide an opportunity to check for differences in reported susceptibility in instances where high agreement should be present.

To further explore how observed susceptibility compares between various antimicrobials against the same submitted sample, an agreement matrix was produced showing the proportion of instances where pairs of drugs arrived at the same susceptibility interpretation. For this matrix, susceptibility was dichotomized to susceptible and non-susceptible (resistant and intermediate combined). Only samples tested against the complete list of drugs in the matrix were included. This list of drugs was compiled based on the most frequent panel tested for the given pathogen and source observed in the data. All drugs in the panel were matched pairwise and the proportion of times the same susceptibility interpretation was observed was recorded in the matrix. A proportion of 1 would indicate that on every sample tested the two drugs always resulted in the same dichotomized susceptibility interpretation. Two agreement matrices were produced, one for *E. coli* isolated from urine samples and one for *S. pseudintermedius* isolated from skin samples.

## 3. Results

The dataset was comprised of 25,147,300 single antimicrobial susceptibility tests performed on 1,295,480 isolates submitted for 760,157 individual patients. A breakdown of high-level descriptive statistics is displayed in Table 1.

**Table 1:** Descriptive breakdown of data used in assessing resistance.

		*E. coli*		*S. pseudintermedius*	
		Amoxicillin (Urine^1^)	Cephalosporin (III)	Cephalosporin (I) (Skin^2^)	Methicillin/Oxacillin
Sample Size (*n*)					
	2019	57,466	94,027	21,404	68,761
	2020	66,537	105,645	23,364	79,846
	2021	71,440	115,367	27,243	92,888
*n* by Species					
	Canine	145,156	243,261	70,985	234,667
	Feline	50,287	71,778	1,026	6,828
*n* by State (All years)					
	Minimum	143 (WY)	238 (WY)	13 (WY)	100 (WY)
	Maximum	44,756 (CA)	66,895 (CA)	12,182 (CA)	43,277 (CA)
	Median	1,582	2,582	625	2,036
	n < 30	0	0	2	0
% Non-susceptible by Species					
	Canine	27.8%	17.9%	41.7%	32.4%
	Feline	28.0%	11.2%	46.4%	43.8%
% Non-susceptible by State					
	Minimum	20.5% (MO)	8.58% (WY)	30.8% (MN)	22.1% (WY)
	Maximum	43.1% (LA)	28.1% (LA)	51.1% (TX)	42.6% (TX)
	National average	33.0%	16.5%	41.9%	32.7%
Relative risk					
	Minimum	0.54 [MO (2021)]	0.41 [ND (2020)]	0.34 [RI (2019)]	0.46 [RI (2019)]
	Maximum	1.39 [AL (2021)]	1.86 [MS (2020)]	1.35 [TX (2019)]	1.42 [TX (2019)]

^1^Urinary samples defined as urine collected by means of catheter, cystocentesis or free catch.

^2^Skin samples defined as samples classified as dermatitis, skin or skin tissue.

### 3.1. Amoxicillin resistant *Escherichia coli* (urinary isolates)

**Figure 1:**
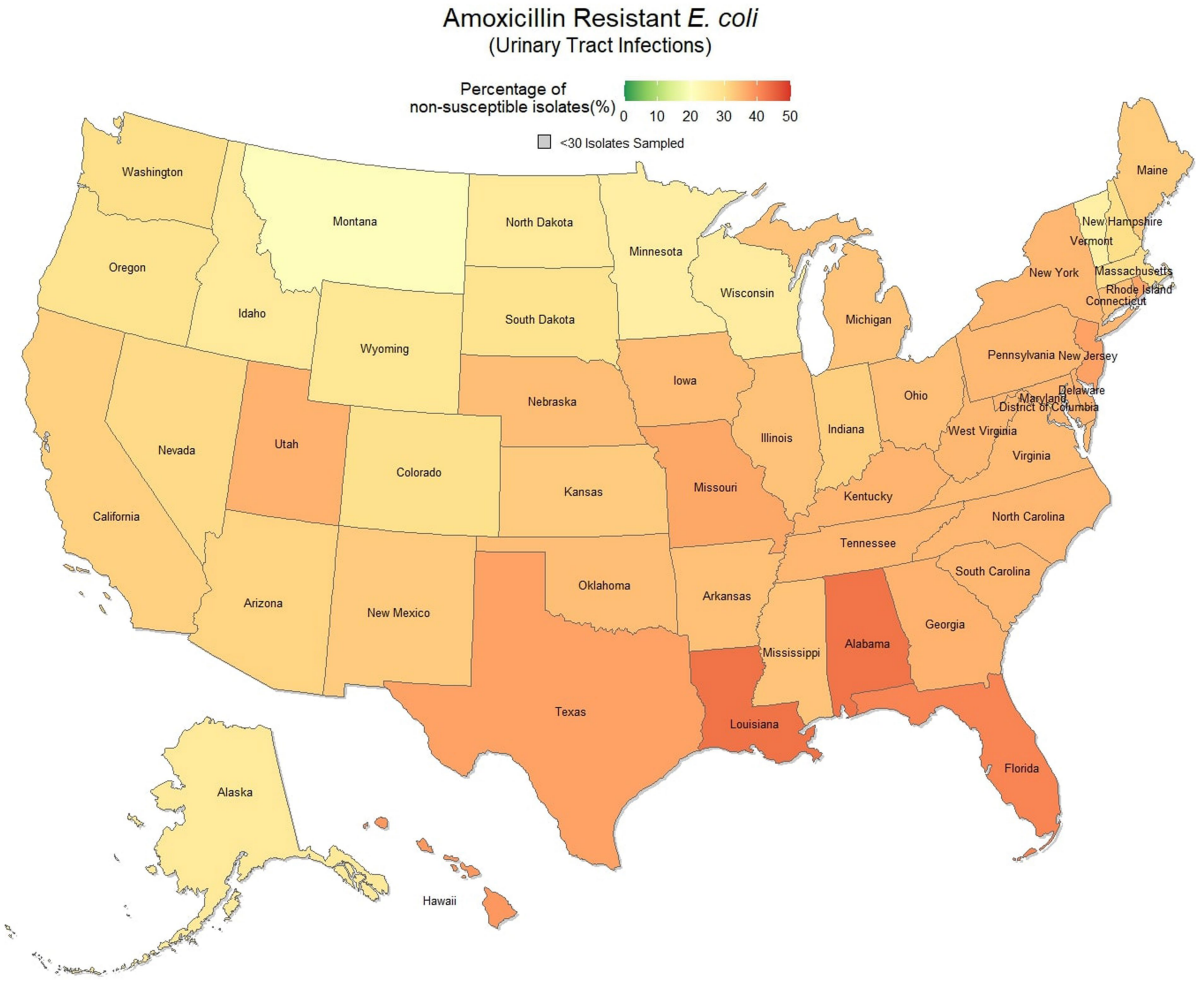
Map of the United States, depicting the percentage of *E. coli* isolates, from urinary tract infections in cats and dogs, found to be resistant to amoxicillin between 2019 and 2021.

**Figure 2:**
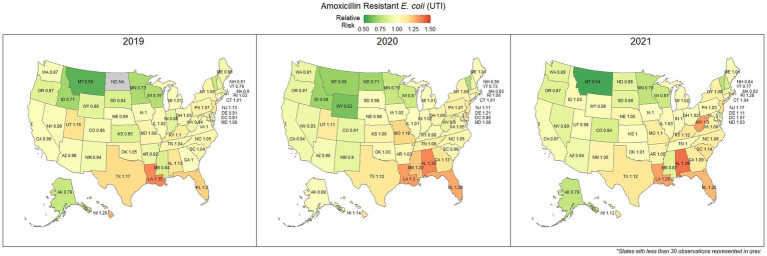
Maps of the United States, depicting the relative risks of amoxicillin resistant *E. coli*, from urinary tract infections in cats and dogs observed in 2019, 2020, and 2021.

Overall, 67.0% (66.8, 67.1%) of *E. coli* were not susceptible to amoxicillin. The lowest percentage of susceptible samples across the three-year study period were observed in Louisiana [56.9% (54.4, 59.5%)] and Alabama [57.0% (53.3, 60.5%)]. The state with the greatest observed susceptibility was Montana [79.4% (75.2, 83.7%)]. A state breakdown of the observed non-susceptible samples can be seen in Figure 1. In general, states in the southeast appear to have observed the greatest proportion of non-susceptible samples of *E. coli* to amoxicillin. Observing the relative risks of each state for each of the 3 years shows relative temporal stability in the distribution of amoxicillin resistant *E. coli* (Figure 2). The southeastern states were continuously observed to have the greatest relative risk each year, in contrast to the western and Midwest states who showed continuous relative risk values at or below one. The range of relative risk (0.54–1.39) indicates a moderate discrepancy in risk across the country.

### 3.2. First-generation cephalosporin resistant *S. pseudintermedius* (skin isolates)

**Figure 3:**
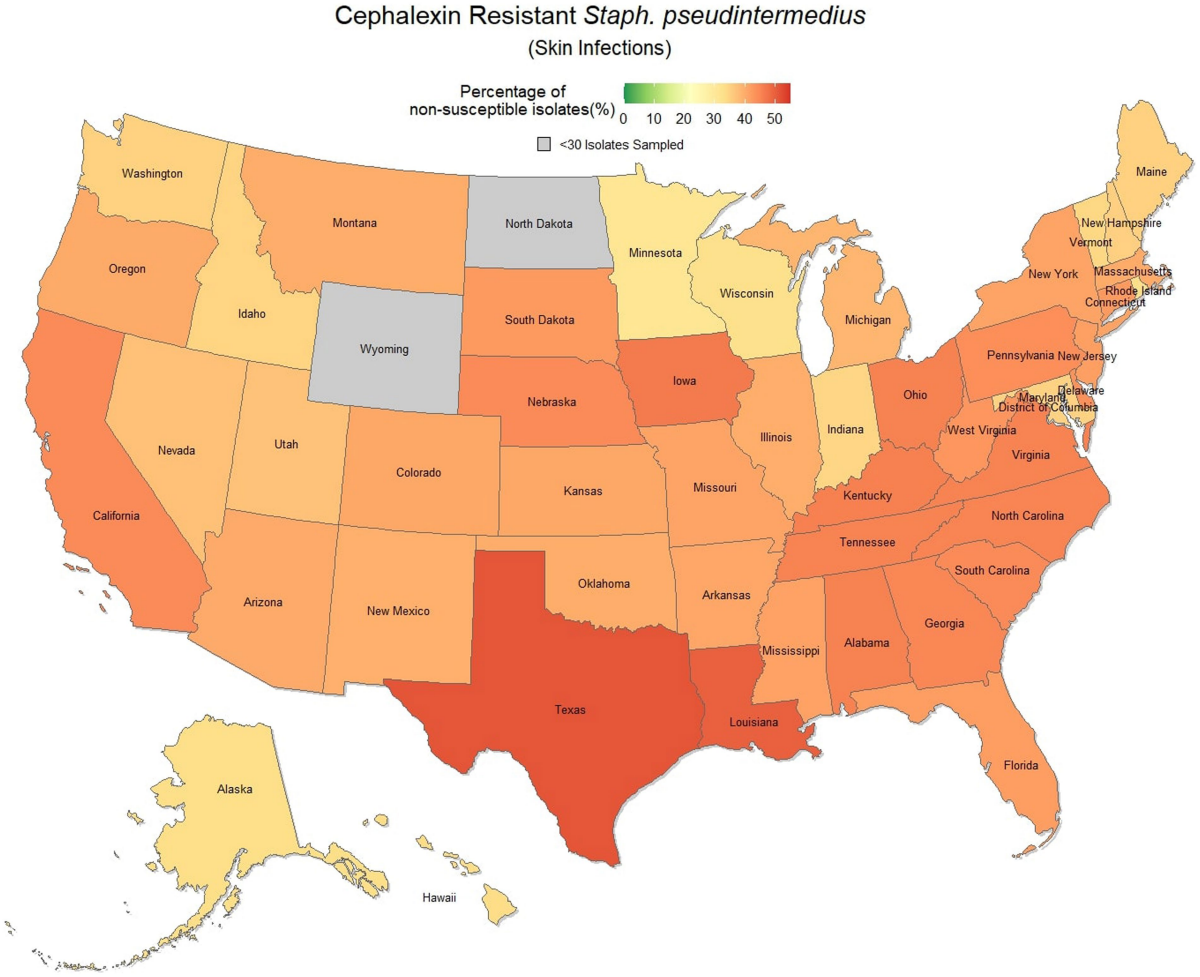
Map of the United States, depicting the percentage of *S. pseudintermedius* isolates, from skin infections in cats and dogs, found to be resistant to cephalexin between 2019 and 2021.

**Figure 4:**
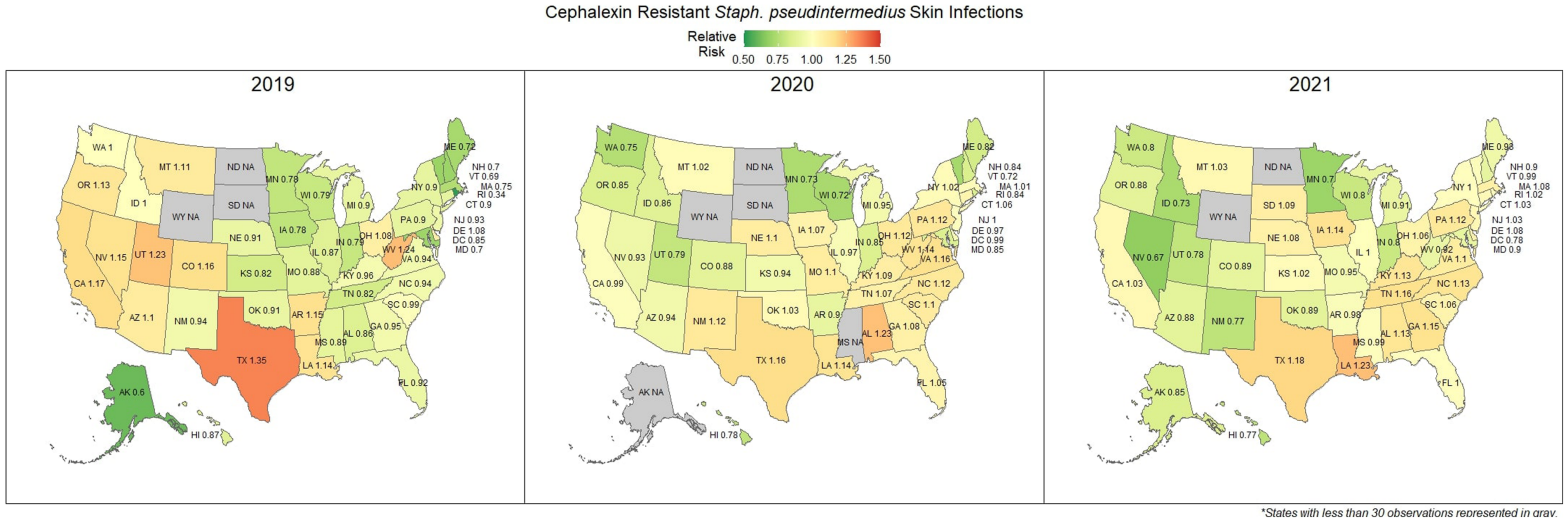
Maps of the United States, depicting the relative risks of cephalexin resistant *S. pseudintermedius*, from skin infections in cats and dogs observed in 2019, 2020, and 2021.

Overall, there was a prominent level of first-generation cephalosporin resistant *S. pseudintermedius* observed across the country, with only 58.1% (57.7, 58.4%) of samples found to be susceptible. Susceptibility as low as 48.8% (47.5, 50.1%) was observed in Texas (Figure 3). Two states, Wyoming and North Dakota, failed to reach the minimum sample size to be included in the map. Mapped relative risks showed a relatively even distribution of risk across the country and stability over the 3 years studied. No clear spatial trend could be concluded through visual analysis (Figure 4).

### 3.3. Third-generation cephalosporin resistant *Escherichia coli* (All sources)

**Figure 5:**
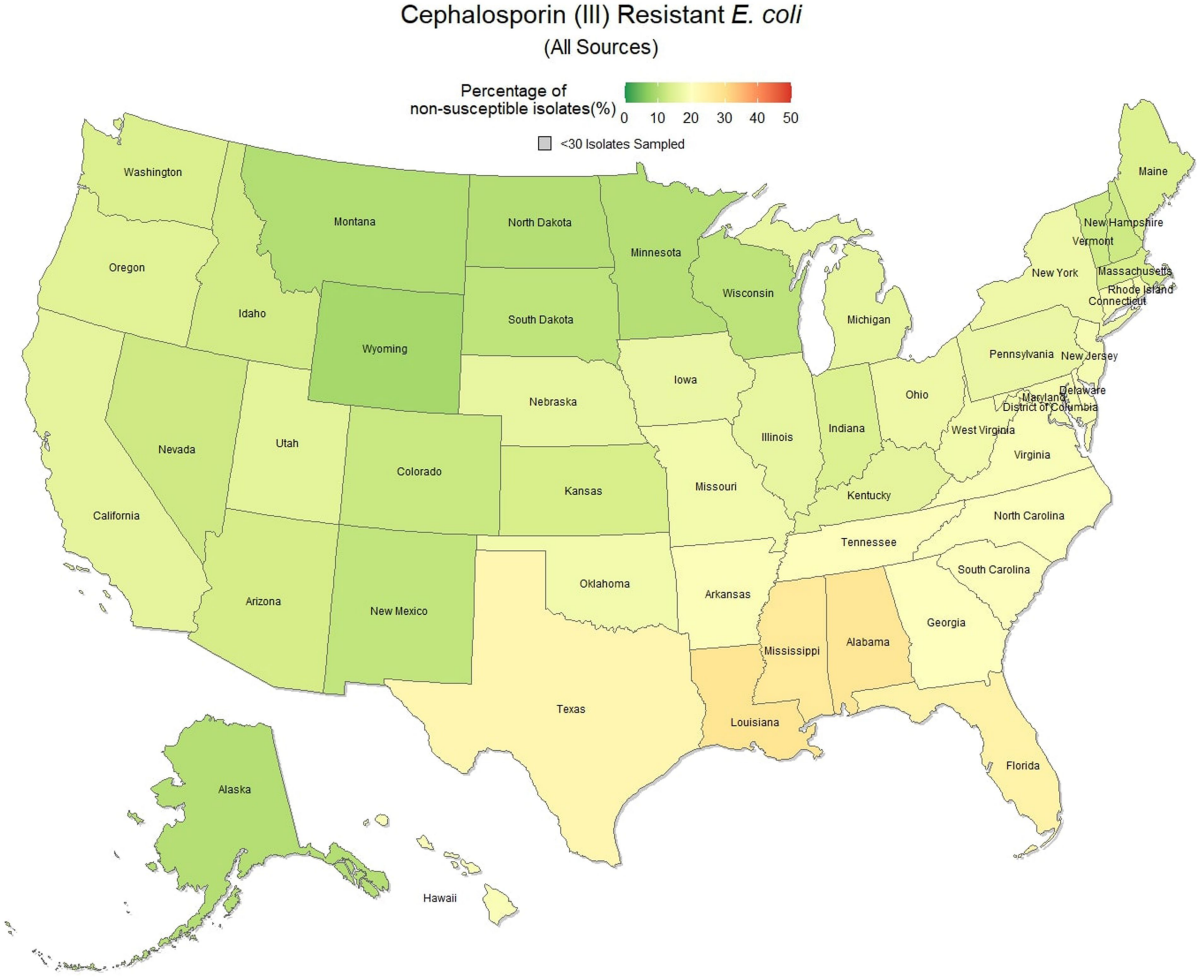
Map of the United States, depicting the percentage of *E. coli* isolates from cats and dogs found to be resistant to cephalosporins (III) between 2019 and 2021.

**Figure 6:**
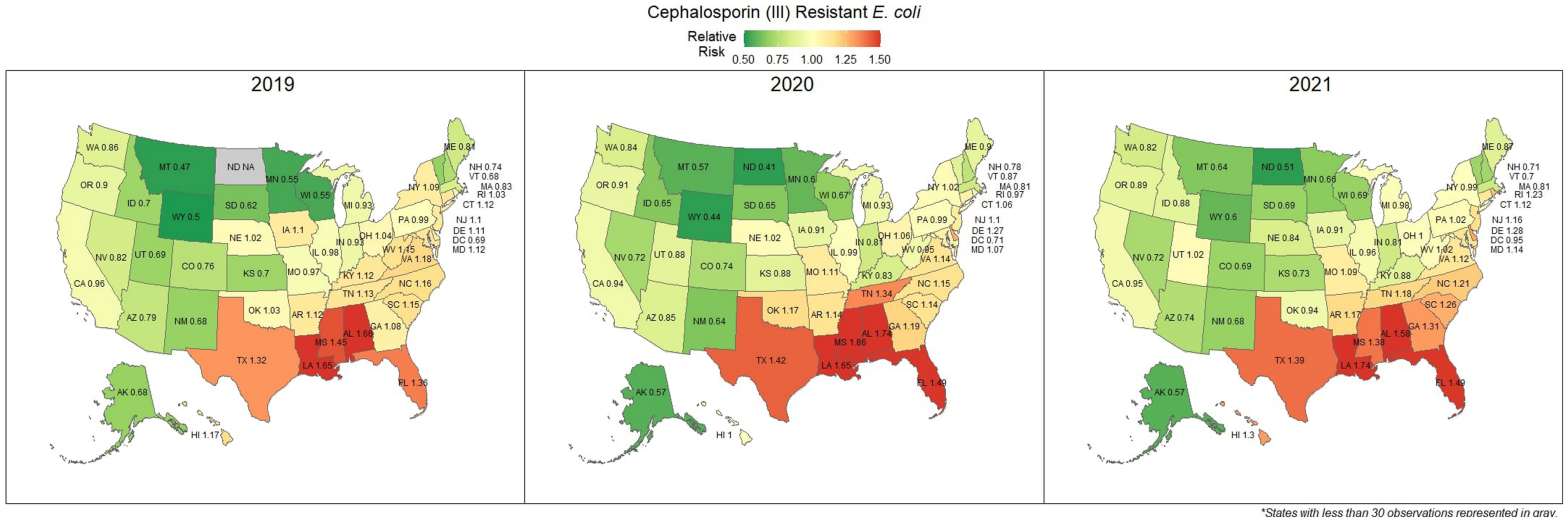
Maps of the United States, depicting the relative risks of cephalosporin (III) resistant *E. coli* infections in cats and dogs observed in 2019, 2020, and 2021.

*E. coli* isolated and tested during the study period were found to be highly susceptible to third generation cephalosporins (cefotaxime, ceftazidime, and cefpodoxime), at 83.5% susceptibility (83.3, 83.6%) across the country (Figure 5). Despite the overall high susceptibility, considerable variability in relative risk was observed, where the southeastern states (Alabama, Florida, Louisiana, Mississippi, and Texas) were up to 1.86 times more likely to observe a resistant sample than the national average (Figure 6). Visual analysis of the relative risk maps revealed a clear spatial pattern of elevated risk in the southeastern states with diminishing risk moving to the northwest.

### 3.4. Methicillin resistant *S. pseudintermedius* (All sources)

**Figure 7:**
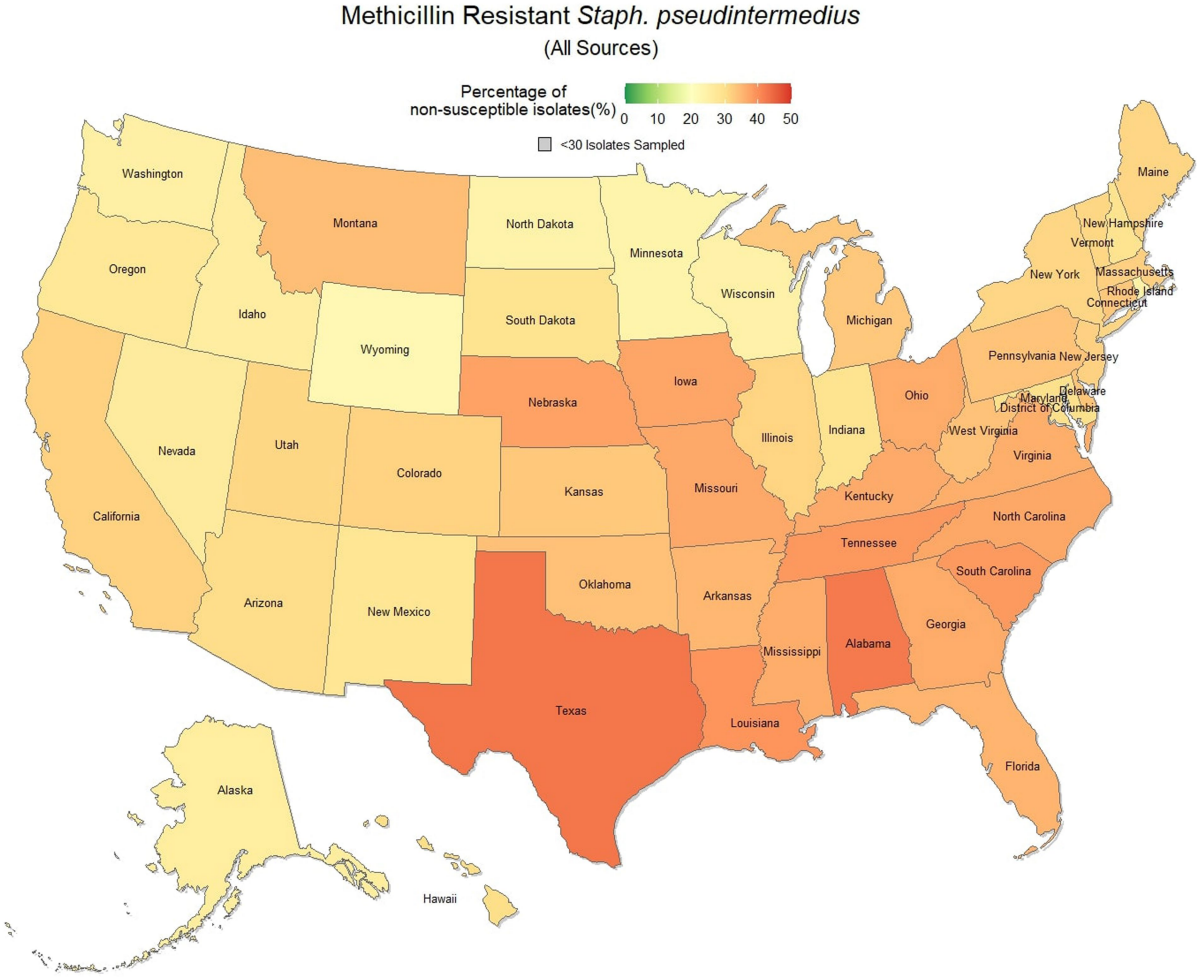
Map of the United States, depicting the percentage of *S. pseudintermedius* isolates from cats and dogs found to be resistant to methicillin/oxacillin between 2019 and 2021.

**Figure 8:**
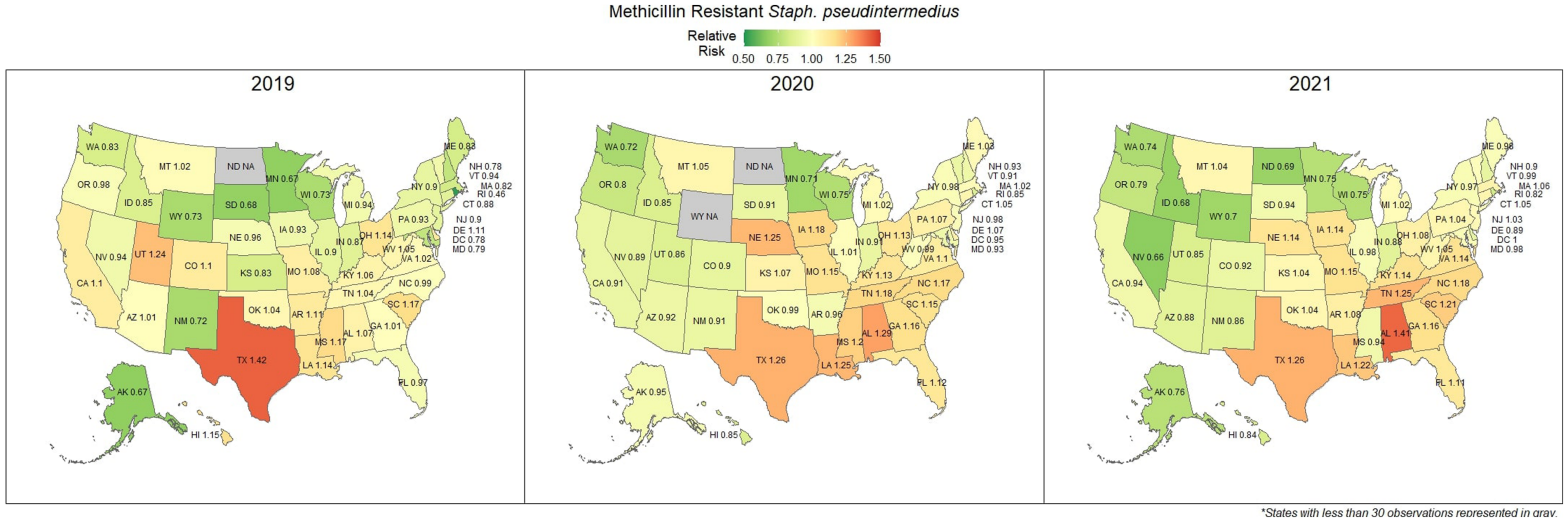
Maps of the United States, depicting the relative risks of methicillin/oxacillin resistant *S. pseudintermedius* infections in cats and dogs observed in 2019, 2020, and 2021.

Methicillin resistant *S. pseudintermedius* was observed across the country, with a national susceptibility rate of 67.3% (67.1, 67.4%). The lowest susceptibility was observed in Texas [57.3% (56.6, 58.2%)] and the highest in Wyoming [77.9% (69.1, 86.7%)]. Similar spatial patterning was observed to the previous scenarios examined, with a greater level of resistance in the southeastern states and Texas, and lower levels of resistance moving to the northwest (Figure 7). Examination of the relative risk maps provide more evidence for this spatial trend and saw consistent distribution patterns over all 3 years (Figure 8). Relative risk values ranged from 0.46, in Rhode Island, to 1.42, in Texas.

### 3.5. Drug susceptibility agreement

**Figure 9:**
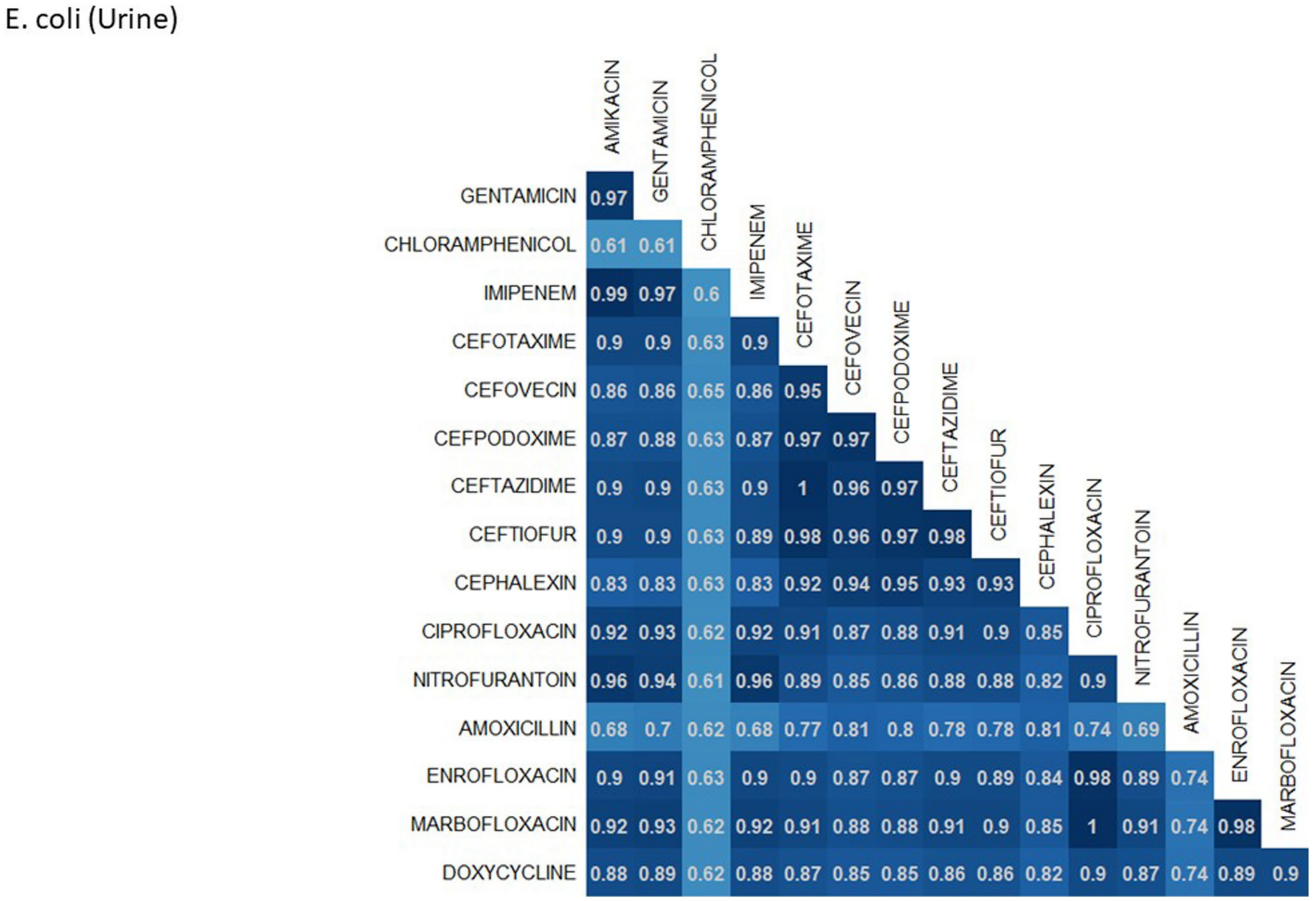
Probability matrix for observing the same AST interpretation between pairwise drug combination in *E. coli* isolated from urine samples from cats and dogs.

**Figure 10:**
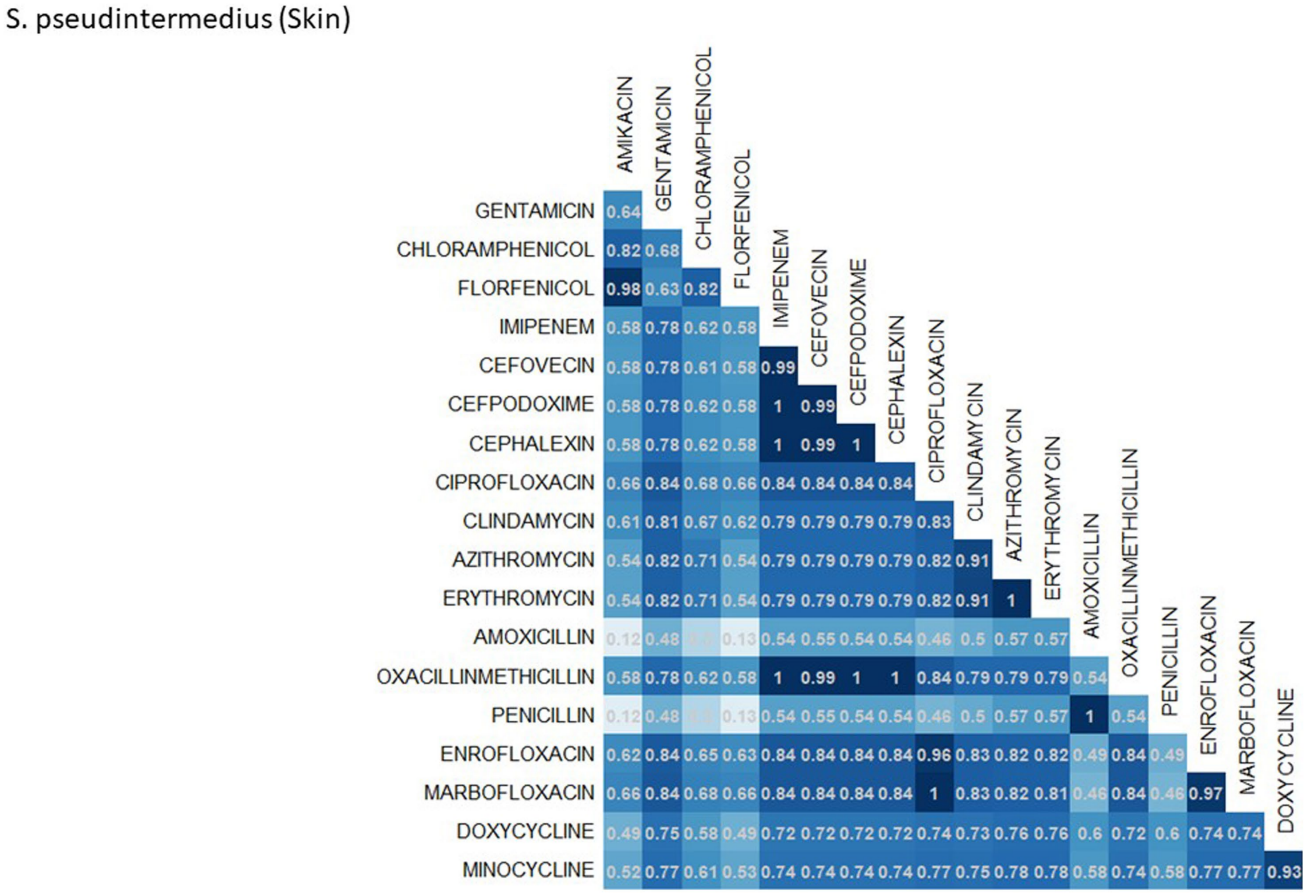
Probability matrix for observing the same AST interpretation between pairwise drug combination in *S. pseudintermedius* isolated from skin samples from cats and dogs.

A breakdown of susceptibility test results for both *E. coli* and *S. pseudintermedius*, against all drugs tested in the third generation cephalosporin and fluoroquinolone classes is presented in Table 2. Two drugs, cefotaxime and ceftiofur failed to meet the minimum sample size requirement to be included. Pradofloxacin, although meeting the CLSI recommended minimum sample size, was removed from the table for *E. coli* due to substantially fewer observations than the other drugs in the same class. Ceftazidime was removed for the same reason for *S. pseudintermedius.* All drugs within the same class were found to possess reasonably similar proportions of susceptible, intermediate and resistant observations (Table 2). The agreement matrices for *E. coli* and *S. pseudintermedius* susceptibility against commonly tested drugs are presented in Figures 9, 10, respectively. A high level of intra class agreement was observed in the matrices. Likewise, drugs acting through similar biological mechanisms showed a high level of agreement, as anticipated.

**Table 2:** Comparison of susceptibility test results across drugs of the same class.

	*E. coli*				*Staphylococcus spp.*			
	Sus.	Res.	Int.	*n*	Sus.	Res.	Int.	*n*
Cephalosporins (III)								
Cefotaxime	88.2%	11.0%	0.9%	322,839	–	–	–	15
Cefovecin	82.1%	16.8%	1.1%	331,080	63.9%	33.1%	3.0%	325,240
Cefpodoxime	83.4%	16.5%	0.1%	331,074	67.0%	32.4%	0.6%	325,067
Ceftazidime	86.0%	12.8%	1.3%	331,073	–	–	–	840
Ceftiofur	85.7%	13.2%	1.1%	315,762	–	–	–	29
Fluoroquinolones								
Ciprofloxacin	90.4%	9.5%	0.1%	331,169	69.8%	29.6%	0.6%	324,939
Enrofloxacin	88.1%	9.5%	2.5%	331,162	65.9%	28.0%	6.0%	325,282
Marbofloxacin	90.4%	9.3%	0.3%	331,168	69.7%	29.6%	0.6%	325,297
Pradofloxacin	–	–	–	41	70.6%	22.4%	7.1%	15,896

## 4. Discussion

The analysis performed in this study, while not comprehensive, offers foundational knowledge regarding the applications of laboratory susceptibility testing data, in addition to the status of several frequently referenced problems of concern in companion animal AMR. While there have been studies on companion animal AMR elsewhere, there has been a paucity of research in the United States (22–24). Many of the presented estimates of the national resistance burden are among the earliest to be reported at a national level for the United States, offering a unique opportunity to compare observed resistance between states under near identical submission and testing conditions. These results found similar spatial patterns of resistance across three of the four drug-pathogen combinations explored, wherein the southeastern states, and Texas were found to have higher than average levels of non-susceptible isolates. Conversely, the more northern states, particularly those to the west, were more likely to see higher levels of susceptibility. This pattern may reveal a spatial trend which should be further assessed to determine causality. In some scenarios (third generation cephalosporin resistant *E. coli* in particular) this pattern was quite pronounced with a range in relative risk of 0.41 in North Dakota to 1.86 in Mississippi, indicating a near 150% difference in risk between the northern and southern states. This pattern indicates that some outside factor of resistance is likely to be present, be it environmental (due to differing climates), cultural (differences in antimicrobial treatment practices), or systematic (a difference in how clinicians are deciding to submit a sample for testing).

Amoxicillin is a common first line drug for sporadic bacterial cystitis in companion animals (25). Similarly, cephalexin (a first-generation cephalosporin) is a commonly prescribed first line drug for the treatment of *S. pseudintermedius* and other skin and soft tissue infections (26). At a national level, resistance to amoxicillin in *E. coli* (urine) and cephalexin in *S. pseudintermedius* (skin) was observed in 33.0 and 41.9% of samples submitted, respectively. These results suggest that a large amount of common urinary and skin infections are resistant to frequently administered first line antimicrobials. Resistance to these first line treatments is a documented concern (27, 28), with susceptibility to amoxicillin in *E. coli* as low as 53% being reported in previous lab datasets in Kansas, United States (29). Resistance estimates may be overinflated in this and other laboratory datasets, as discussed subsequently in more detail. Patients included in antimicrobial susceptibility testing (AST) datasets are more likely to have failed first line treatments, thus warranting testing to find a suitable replacement. However, the general spatial patterning should remain representative, where resistance appears to be more prevalent further into the southern states. A particular pattern of interest was the noticeable switch in relative risk between the east and west coast states for cephalexin resistant *S. pseudintermedius* occurring between 2019 and 2021, for which no explanation can be offered.

*E. coli* and *S. pseudintermedius* both have the potential to cause zoonotic infections. *E. coli* is a common bacterium found in the gastrointestinal tracts of mammals. This bacterium can be spread through contact with infected fecal matter, causing a range of symptoms, from mild to severe. Several studies have highlighted the zoonotic potential of *E. coli* from companion animal reservoirs, including strains resistant to common antibiotics (30–33). *S. pseudintermedius* is an opportunistic pathogen frequently observed on the skin and mucosa of canines, and to a lesser extent, felines. While often a harmless component of the natural flora, *S. pseudintermedius* may present a zoonotic threat to humans who come into contact with infected companion animals (13, 34). The zoonotic potential of both of these bacteria underscore the importance of monitoring for antimicrobial resistance in companion animals which could subsequently spillover into humans and vice versa.

Increasing awareness of multidrug resistant strains of *E. coli* and *S. pseudintermedius* has raised concern for the health of humans and animals (35, 36). Extended spectrum beta lactamases (ESBL) confer resistance to multiple drug classes including third generation cephalosporins, and is becoming more commonly reported in *E. coli* isolates in companion animals (37). Susceptibility to third generation cephalosporins in *E. coli* samples analyzed in this study appears to be high, with a national average of 83.5% of samples showing sensitivity to these drugs. This level was observed consistently across all third-generation cephalosporin drugs tested against *E. coli.* Of notable concern is the discrepancy in resistance between the southern and northern states, as seen in Figure 6. Comparison of relative risk values between these states indicates that a grouping of elevated resistance appears to exist in the southeastern states and should be further assessed. Methicillin resistant *S. pseudintermedius* presents another growing concern for zoonoses and clinical outbreaks in companion animals (38). Looking at all laboratory susceptibility results for *S. pseudintermedius* against methicillin (oxacillin) within the United States between 2019 and 2021 confirms that considerable levels of resistance are being observed in companion animal isolates. On average 32.7% of isolated *S. pseudintermedius* were classified as non-susceptible to methicillin, about ten percentage points lower than the resistance observed to first- generation cephalosporins. Methicillin and first-generation cephalosporins were expected to show greater similarity in their levels of resistance, given that methicillin resistant *S. pseudintermedius* will typically also express resistance to other beta lactams, including cephalexin (39). In the drug agreement matrix (Figure 10), this relationship is observed, as cephalexin and methicillin (oxacillin) showed perfect agreement on all *S. pseudintermedius* samples when tested in parallel (cephalexin and oxacillin both tested on the same isolate). However, when isolates tested against cephalexin, oxacillin or both are included, as is with the mapped state-level resistance, differences were observed. This discrepancy illustrates how this data can be perceived differently based on the inclusion and exclusion criteria and methodologies applied. A three-fold difference in sample size between *S. pseudintermedius* resistance testing against oxacillin and cephalexin may explain this observed discrepancy.

Once more, the resistance observed in a laboratory diagnostics setting is likely to be greater than in the general population, but nevertheless, these findings indicate that MRSP, and to a lesser extent ESBL, are present issues in companion animals of the United States. Higher levels of resistance to first line and subsequent treatments could lead to higher instances of extra-label antimicrobial usage, wherein drugs reserved for human infections are used in companion animals to treat difficult infections (4). Extra-label usage of these drugs may increase the risk of emergence of infections in companion animals resistant to high profile antibiotics in humans such as carbapenems for cephalosporin resistant *E. coli.*

Electronic health records and databases of laboratory antimicrobial susceptibility testing offer the benefit of widespread data coverage as well as consistency in methods to make comparisons over space and time. The results from hundreds of thousands of susceptibility tests, conducted each year, offer interminable information from which numerous hypotheses can be developed and tested at scale, without the need for additional financial resources and patient recruitment. Many epidemiological studies in AMR would be otherwise infeasible, especially those looking to cover a large study area or period of time. This quantity of data points opens the possibility for specialized analysis methods over time and space, which often require sizeable amounts of data but can produce results that cannot be seen at smaller scales. Time series and spatial epidemiology do not answer the question of ‘how’ but rather highlight the ‘where’ and ‘when’ which can be used to allocate resources, direct future research and serve as surveillance tools for early detection of an outbreak.

Further opportunities exist to leverage ongoing laboratory testing into dynamic data visualizations and dashboards for surveillance and decision making. As more samples are processed each day, plots and figures can be continuously updated such that the information is always up to date, as opposed to static plots which can become outdated within a year’s time. By involving data science experts, these various information systems at the regional and national level can be made to integrate with one another, allowing for data sets to be combined. Interactive dashboards have become a popular tool in epidemiology to house data visualizations and offer the added functionality of dynamically filtering datasets into specific information pertinent to a given scenario. In this case, the multitude of drug/pathogen/source combinations makes antimicrobial resistance an excellent candidate for this type of tool, one that can be leveraged by researchers for hypothesis generation, as well as clinicians for guiding of antimicrobial treatment decisions. Furthermore, dashboards can be made to automatically receive, process and publish new data with little need for human involvement. The ability to filter down to a specific region, infection site, pathogen, and drug combination could be an invaluable tool, however, could also pose significant risk to antimicrobial stewardship efforts if not thoughtfully produced. Failure to recognize the limitations and biases of AST datasets, and a fundamental understanding of how they may differ from the status of the general population could see veterinarians reaching for higher priority antimicrobials more frequently if first line treatments are represented as uncommonly effective. Clinicians should be involved in the dashboard development process to understand how these tools will be perceived and how the data will be used to make informed decisions.

Although laboratory susceptibility testing presents many opportunities for research into the epidemiology of antimicrobial resistance, several notable data concerns exist and must be assessed. Data such as these contain large amounts of information regarding patient, location and pathogen factors, but two key pieces of information that are not present are the reason why culture specimens were submitted and whether there was prior antimicrobial treatment of that infection (i.e., testing in response to treatment failure). Susceptibility testing is a key part of proper antimicrobial stewardship practices but is not always feasible due to time or cost constraints, or pressure from the client to prescribe treatment quickly. As a result, clinicians may often prescribe a common first line antibiotic, and only submit a sample for testing if that treatment fails, in order to find a suitable replacement. This inherently results in the sampled population of these datasets being representative of more resistant pathogens than in the general population due to sampling bias. However, the extent of this bias is unclear and with limited information regarding why the test was ordered it can be difficult to assess and correct. Further work should seek to understand the motivations of clinicians for both testing and prescribing of antimicrobials, with the aim of understanding the extent of bias within these data sources. Estimates of resistance in the present work should be considered as over-estimations until these biases can be adequately assessed. Additionally, large quantities of data can lead to problems in analysis which can require special consideration and methods to overcome. Simple statistical tests will often find significance when using large datasets due to the exceptionally large statistical power. Therefore, practical significance should always be considered even when statistically significant.

Antimicrobial resistance is a complex health issue, firmly situated in the realm and responsibilities of a One Health framework. Historically, companion animals have been neglected in the AMR discourse, due at least in part to the lack of available population level data to conduct epidemiological studies (15). However, this study has demonstrated the usefulness of deidentified commercial laboratory data to assess epidemiological resistance patterns across a large study area. Using these datasets removes a primary barrier to the inclusion of companion animals into One Health studies and offers a missing piece to the puzzle. In addition, these data can be used as a basis for AMR surveillance in the veterinary community and allow for data-driven decision making in empirical therapy. Numerous in-depth analyses will come from access to such data and can be used to monitor known, emerging and novel resistance concerns.

## Data availability statement

The data analyzed in this study is subject to the following licenses/restrictions: privately owned commercial dataset. Requests to access these datasets should be directed to IDEXX Laboratories, Inc.

## Ethics statement

Ethical review and approval was not required for the animal study because this study utilized de-identified secondary data. Written informed consent for participation was not obtained from the owners because this work is in compliance with IDEXX terms and conditions which are agreed upon at the time of test ordering, and all personal information was removed.

## Author contributions

AP and DS: data extraction and preparation. KS, JW, ZP, and TB: analysis and interpretation of results and draft manuscript preparation. All authors were involved in the study conceptualization and design, reviewed the results, and approved the final version of the manuscript.

## Conflict of interest

AP and DS were employed by IDEXX Laboratories, Inc. TB holds the IDEXX Chair in Emerging Technologies and Preventive Healthcare. KS was supported by the IDEXX Chair.

The remaining authors declare that the research was conducted in the absence of any commercial or financial relationships that could be construed as a potential conflict of interest.

## Publisher’s note

All claims expressed in this article are solely those of the authors and do not necessarily represent those of their affiliated organizations, or those of the publisher, the editors and the reviewers. Any product that may be evaluated in this article, or claim that may be made by its manufacturer, is not guaranteed or endorsed by the publisher.
